# Evaluation of Follow-Up and Treatment Results in Coats’ Disease

**DOI:** 10.4274/tjo.12754

**Published:** 2016-10-17

**Authors:** Zafer Cebeci, Şerife Bayraktar, Yusuf Cem Yılmaz, Samuray Tuncer, Nur Kır

**Affiliations:** 1 İstanbul University İstanbul Faculty of Medicine, Department of Ophthalmology, İstanbul, Turkey

**Keywords:** Coats’ disease, laser photocoagulation, cryotherapy, pars plana vitrectomy

## Abstract

**Objectives:**

The aim of this study was to evaluate the clinical features, follow-up and treatment results of patients diagnosed with Coats’ disease.

**Materials and Methods:**

Medical records of 27 patients diagnosed with Coats’ disease in our clinic were reviewed retrospectively. All patients underwent complete ophthalmological examination and fundus photography was taken. Disease stage and treatment methods used were recorded.

**Results:**

Twenty-seven eyes of 27 patients were included in the study. Mean age was 9.03 years; 21 patients were male and 6 were female. Three patients were older than 18 years old. Based on the Shields classification, 1 (3.7%) eye was stage 2A, 4 (14.8%) eyes were stage 2B, 6 (22.2%) were stage 3A1, 3 (11.1%) were stage 3A2, 1 (3.7%) was stage 3B, 4 (14.8%) were stage 4 and 8 (29.6%) were stage 5. Fourteen patients underwent treatment, 12 of whom had combined therapy. The most common treatment modalities were laser photocoagulation and cryotherapy. Encircling band was done in one patient and pars plana vitrectomy in 3 patients. Enucleation was done in 5 patients.

**Conclusion:**

Coats’ disease is a chronic disease and main goal of treatment is to eliminate the vascular anomalies and their complications using repetitive combination therapies. Treatment in the early stages can lead to functional success, and in advanced stages can result in a salvageable eye.

## INTRODUCTION

Coats’ disease is a nonhereditary condition characterized by retinal capillary telangiectasia, arterial aneurism, exudation and exudative retinal detachment.^1^ Coats^2^ first described the disease in a 1908 report of a patient group with retinal telangiectasia with massive intra- and subretinal exudation. In 1912, Leber^3^ reported a condition involving retinal vascular anomalies but without exudation or serous retinal detachment and proposed the pathology may be an early or milder form of Coats’ disease.

Coats’ disease predominantly affects males and is usually unilateral.^1,4^ The condition is often detected in early childhood, but may occasionally appear in adults and shows slower progression with later onset.^4,5^ Shields et al.^6^ divided Coats’ disease into 5 stages ranging from mild disease with retinal telangiectasia only to severe, advanced disease with a blind, painless eye and possible cataract and phthisis bulbi (Table 1).

Treatments such as laser photocoagulation, cryotherapy and intravitreal corticosteroid or anti-vascular endothelial growth factor (anti-VEGF) injection may be effective against telangiectatic vasculature in the early stages of the disease.^1,7^ More advanced disease with extensive exudative retinal detachment may also require additional surgical interventions such as vitrectomy, scleral buckling, and external drainage.^1,7^

The aim of this study was to examine the clinical characteristics and follow-up and treatment outcomes of patients diagnosed with Coats’ disease in our clinic.

## MATERIALS AND METHODS

We retrospectively analyzed the charts of patients diagnosed with Coats’ disease in our clinic. Diagnosis of Coats’ disease was based on idiopathic telangiectasia, aneurysms, intraretinal and/or subretinal exudation or exudative retinal detachment in the absence of any other ocular disease. Patients with idiopathic juxtafoveal telangiectasia, other pathologies which lead to retinal exudation, or intraocular inflammation were excluded from the study.

Patients’ age, gender, complaints at presentation, disease laterality, and follow-up time were recorded. Best corrected visual acuity, intraocular pressure, and slit-lamp and fundoscopic examination findings at initial and final examinations were also recorded. Fundus photography was performed in suitable patients; images were acquired with a RetCam system (Clarity Medical Systems, Pleasanton, CA, USA) for children examined under general anesthesia, and with a Zeiss FF450 plus (Carl Zeiss, Meditec, Dublin, CA, USA) in other patients. Fundus fluorescein angiography (FFA) was performed when possible. Treatments applied during follow-up were recorded in detail. The study was designed and conducted in accordance with the principles of the Declaration of Helsinki.

## RESULTS

The study included 27 eyes of 27 patients diagnosed with Coats’ disease; 21 patients (77.8%) were male, 6 (22.2%) were female. Mean age at presentation was 9.03 years (range, 18 months-44 years). Three patients (11.1%) were over the age of 18 at presentation. Mean follow-up time was 69.5 months (range, 6-324 months). All patients had unilateral involvement, 15 (55.6%) in the right eye and 12 (44.4%) in the left eye. Of the 18 patients referred to our clinic for advanced testing and treatment, referral diagnosis was intraocular tumor in 10 (55.6%), Coats’ disease in 2 (11.1%), uveitis in 2 (11.1%), cataract in 1 (5.6%), retinal detachment in 1 (5.6%) and strabismus in 1 (5.6%). None of the patients had a family history of similar ocular pathology. Fourteen patients (51.8%) had low vision, 14 (51.8%) strabismus, 8 (29.6%) leukocoria and 1 (11.1%) eye pain. Mean age was 13.5 years in patients presenting with low vision, 5.5 years in patients presenting with strabismus and 3.7 years in patients with leukocoria.

**Figure 1:**
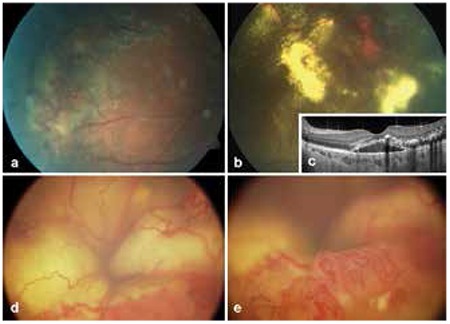
Images from various stages of Coats’ disease, a) peripheral telangiectatic vessels and exudation in stage 2A disease, b) telangiectatic vessels and exudation in stage 2B, c) optical coherence tomography image from the patient in Figure 1b showing subretinal fluid and hyperreflective exudation at the macula, d) image from a patient referred for intraocular tumor, stage 4 disease with total retinal detachment, e) Inferior peripheral telangiectatic vessels of patient in Figure 1d

At initial presentation, disease severity was stage 2A in 1 eye (3.7%), stage 2B in 4 eyes (14.8%), stage 3A1 in 6 eyes (22.2%), stage 3A2 in 3 eyes (11.1%), stage 3B in 1 eye (3.7%), stage 4 in 4 eyes (14.8%) and stage 5 in 8 eyes (29.6%) (Figure 1).

FFA was done in 13 patients and showed filling of telangiectatic vessels, bulb-like hyperfluorescence and avascular regions in the early phase and diffuse hyperfluorescence due to leakage from telangiectatic vasculature in the late phase.

**Figure 3:**
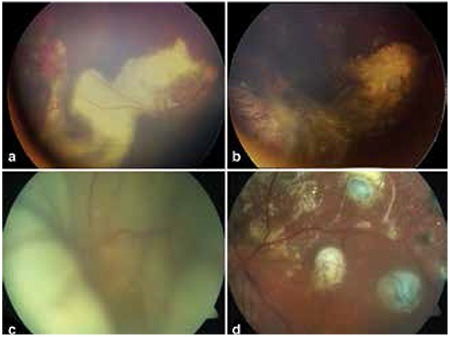
Fundus photographs taken before and after treatment, a) Telangiectatic vessels in the temporal periphery and exudation extending to the macula in a patient with stage 2B Coats’ disease, b) Post-treatment image from the same patient showing cryo scars, fibrotic nodule at the macula and exudation following cryotherapy, c) Pre-treatment image from a patient with total retinal detachment, d) Image from the same patient following pars plana vitrectomy, internal drainage, endophotocoagulation and endocryotherapy

Patient characteristics and treatments administered are shown in Table 2. Coats’ disease was treated with combined therapy in 12 patients and with a single treatment modality in 2 patients. In 13 patients, aneurysms and telangiectases were treated with laser photocoagulation therapy repeated at specific intervals. Nine patients were treated with cryotherapy (Figures 3a and b), 3 with intravitreal bevacizumab injection, 2 with intravitreal triamcinolone acetonide injection and 1 with intravitreal dexamethasone implant. One patient was treated surgically with scleral buckle and 3 patients underwent pars plana vitrectomy (PPV) (Figures 3c, 3d). PPV with internal tamponade was performed in 2 patients (#8 and 13) due to the development of total retinal detachment during follow-up and in the other patient due to subtotal retinal detachment with macular involvement at initial presentation. None of the patients treated for Coats’ disease required enucleation during follow-up. Enucleation was performed in 3 patients due to neovascular glaucoma and painful eye and in 2 patients because of phthisis. Follow-up without treatment was recommended in the remaining 8 eyes due to lack of light perception or pain.

## DISCUSSION

Coats’ disease is a disorder of unknown etiology that typically features idiopathic retinal telangiectases, aneurysms and retinal exudation leading to vision loss.^1^ Although some cases of Coats’ disease are asymptomatic, most patients diagnosed in childhood present with leukocoria, strabismus or low vision.^6^ Shields et al.^4^ evaluated a series of 150 cases and determined that 34% presented with low vision, 23% with strabismus and 20% with leukocoria. In a study including 97 eyes, approximately half presented with low vision, followed by strabismus and leukocoria.^8^ The most common complaints at presentation in our series (reported by about half of our patients) were low vision and strabismus. Low vision was particularly common in older Coats’ patients, whereas strabismus and leukocoria were the most common complaints at presentation in early childhood, likely due to children’s inability to describe their vision problems or their families not recognizing them.

Coats’ disease is usually seen in children, though there are cases with adult onset. Smithen et al.^5^ reported Coats’ disease in 13 patients with a mean age of 50 years. Although these patients exhibit symptoms similar to those in children, the authors stated that disease detected in older patients followed a slower progression than seen in children and that this may lead to a later diagnosis. A community-based study demonstrated that stage 3 or higher disease was more common at younger ages, while early stages were more common in older patients.^9^ In the present study, 3 of 27 patients were over 18 years of age. Two of those patients were followed with repeated treatment and the other patient was followed without treatment because the eye was blind.

Telangiectatic and aneurysmal vessels, particularly in the periphery, may be accompanied by avascular areas which can be visualized with FFA.^1,7^ In one reported series of adult Coats’ patients, areas of capillary nonperfusion were observed in regions of vascular abnormalities in 91.7% of cases.^5^ Avascular fields and vascular anomalies may appear on FFA in fellow eyes without ophthalmoscopic findings as well as the involved eye.^10^ It has been demonstrated that intraoperative FFA-guided laser photocoagulation may be more effective when applied in the early disease stages and that this may reduce the number of repeat treatments.^11^ FFA is a particularly useful auxiliary imaging modality in terms of identifying areas of nonperfusion during treatment and monitoring the regression of vascular anomalies during follow-up.

The primary treatment for Coats’ disease is ablation of the vascular anomalies that cause exudation, thus reducing intraretinal and subretinal exudation and protecting vision and the eye. In patients with stage 2 or 3 disease without severe detachment, laser photocoagulation and cryotherapy have been shown to be effective against telangiectases and aneurysms.^6,12,13,14^ Repeated laser photocoagulation or cryotherapy applications may be required at certain intervals during follow-up. In a study evaluating 17 patients ranging from stage 2A to 4, at the end of follow-up with repeated laser photocoagulation therapy, the globe was conserved in 94% of patients and about half had a final visual acuity of 0.4 or better.^12^

Applying laser photocoagulation and cryotherapy is difficult in the presence of dense exudation. Intravitreal triamcinolone reduces subretinal fluid and exudates when administered as an initial treatment, even in cases of total bullous retinal detachment, and can therefore facilitate the application of other therapies for vascular pathologies.^15,16^ Fifteen patients initially treated with intravitreal 4 mg/0.1 mL triamcinolone injection (with additional subretinal fluid drainage in some cases) all showed reduced size of telangiectatic vessels at 1 month follow-up.^15^ However, it must be kept in mind that intravitreal steroid injection can lead to cataract and glaucoma.^17^

Increasing use of anti-VEGF agents has led to their application in Coats’ disease as well.^18,19,20^ It has been shown that VEGF levels are elevated in the aqueous humour in Coats’ disease and increase significantly as the disease progresses.^21^ Histopathologic examinations have revealed macrophages in the subretinal space and increased vascular permeability caused by these cells, in addition to increased expression of VEGF, which leads to angiogenesis.^22^ Administering 1.25 mg intravitreal bevacizumab before conventional treatments like laser coagulation and cryotherapy has been shown to positively influence visual outcomes.^18^ Villegas et al.^19^ applied intravitreal bevacizumab and laser photocoagulation to 24 advanced stage Coats’ patients with exudative retinal detachment and observed regression of the exudative retinal detachment in all cases. Even in patients with stage 3B and 4 disease, adjuvant or neoadjuvant application of intravitreal ranibizumab may lead to partial visual recovery.^20^ However, there remains the fact that combination therapy with intravitreal anti-VEGF injection may result in vitreoretinal fibrosis and tractional retinal detachment.^23^ Laser photocoagulation and cryotherapy were also the most common treatment modalities in the current case series, especially in earlier stage disease. A subset of our patients received intravitreal corticosteroid or anti-VEGF injections, and all patients underwent repeated treatments. Intravitreal corticosteroid or anti-VEGF injections may be beneficial in reducing exudation in cases where a single treatment method does not yield satisfactory results or to facilitate additional procedures in selected patients.

Vitreoretinal surgery and scleral buckling are preferred for advanced Coats’ disease (stage 3 and 4) patients with tractional bands or proliferative vitreoretinopathy.^1,24,25,26^ Additional internal or external subretinal fluid drainage also facilitates the application of laser photocoagulation or cryotherapy. Mutfuoglu ve Gulkilik^25^ achieved positive anatomic and functional outcomes in 5 patients with PPV, internal subretinal fluid drainage and silicone oil tamponade. Despite postoperative anatomic success, functional success may be achieved in a small number of patients.^1^ Although the operated patients in our study did not show functional improvement, anatomic success was achieved and maintained throughout follow-up.

Some patients present with advanced disease which does not benefit from treatment, while others show disease progression despite treatment. Approximately one in four patients require enucleation due to total retinal detachment and neovascular glaucoma.^6,27,28^ Other than those who underwent enucleation for painful eye or phthisis, none of the patients treated in our study required enucleation during follow-up. However, some patients may have reduced level of vision despite treatment and conservation of the eye.

Other causes of exudative retinal detachment should be considered in the differential diagnosis of Coats’ disease. Retinoblastoma, persistent hyperplastic primary vitreous, Norrie’s disease, and familial exudative vitreoretinopathy in particular are more common in childhood and should definitely be considered.^1^ Although rare in adults, the differential diagnosis should include Eales’ disease, vasoproliferative tumor, idiopathic retinal vasculitis and neuroretinitis, and sickle-cell anemia. About half of the patients referred to our clinic had an initial diagnosis of intraocular tumor. All of these patients could have been correctly diagnosed using ophthalmoscopic examination and additional imaging methods like FFA and ultrasonography when needed. Features such as lack of family history, predominance in males and typically unilateral manifestation, absence of vitreous opacity, presence of telangiectases, and no apparent mass can also assist diagnosis. Computed tomography and magnetic resonance imaging techniques can help differentiate between an intraocular malignancy and advanced stage Coats’ disease.^1^

Coats’ disease is a chronic disease requiring long-term follow-up. Favorable visual outcomes can be achieved with early detection and treatment with combined therapies as required. Even when treatment is not able to restore sight, it is beneficial in saving the eye. It must be noted that new telangiectases and aneurysms can develop over the course of follow-up and may require retreatment.

### Ethics

Ethics Committee Approval: Retrospective study, Informed Consent: It was taken.

Peer-review: Externally peer-reviewed.

## Figures and Tables

**Table 1:**
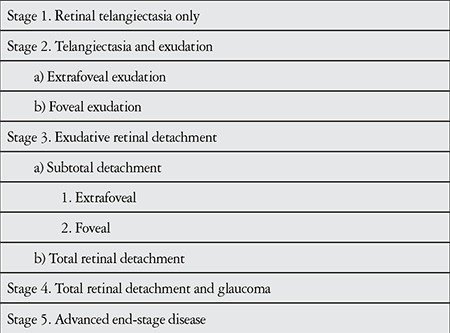
Staging of Coats’ disease

**Table 2:**
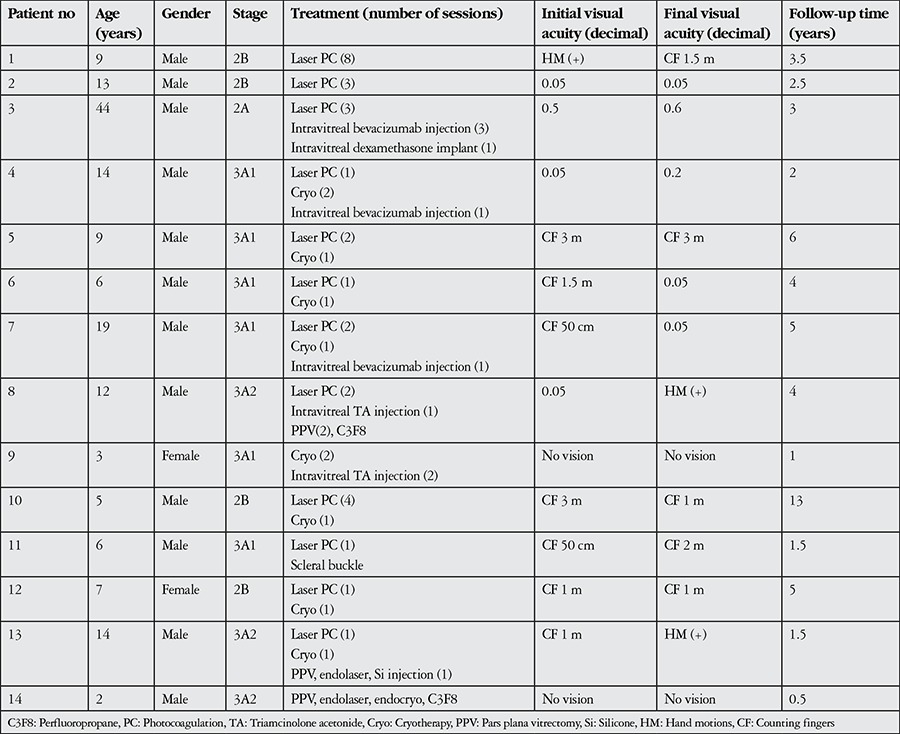
Demographic characteristics, treatments and visual outcomes of patients with Coats’ disease

**Figure 2:**
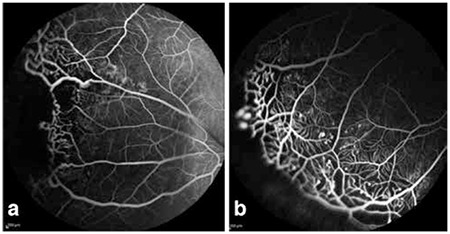
Fundus fluorescein angiography images of Coats’ disease, a) Telangiectatic vessels and avascular areas in the temporal periphery, b) Inferior peripheral image from same patient
